# From *Biomphalaria glabrata* to *Drosophila melanogaster* and *Anopheles gambiae*: the diversity and role of FREPs and Dscams in immune response

**DOI:** 10.3389/fimmu.2025.1579905

**Published:** 2025-04-30

**Authors:** Hongyu Li, Qingzhi Zhao, Jialu Xu, Xianwei Li, Xintong Chen, Yijie Zhang, Hairun Li, Yunhuan Zhu, Mingcheng Liu, Ling Zhao, Dingji Hua, Xiaofen Zhang, Keda Chen

**Affiliations:** ^1^ Key Laboratory of Artificial Organs and Computational Medicine in Zhejiang Province, Shulan International Medical College, Zhejiang Shuren University, Hangzhou, China; ^2^ Ocean College, Beibu Gulf University, Qinzhou, China

**Keywords:** invertebrates, immune molecules, BgFREP, DmDscam, AgDscam

## Abstract

Fibrinogen-related proteins (FREPs) and Down syndrome cell adhesion molecules (Dscams) are important immune-related molecules in invertebrates. Although they are found in different taxonomic groups and possess unique functions, both exhibit high diversity and adaptability. FREPs are characterized by their fibrinogen-related domains and have been primarily studied in mollusks, such as *Biomphalaria glabrata*. Through mechanisms of diversity generation, such as gene conversion and point mutations, *Bg*FREP plays a critical role in the host’s defense against parasites. Dscams are immunoglobulin-like transmembrane proteins, mainly studied in arthropods, such as *Drosophila melanogaster* and *Anopheles gambiae*. Through alternative splicing, Dscams generate multiple isoforms that participate in pathogen recognition and the precise wiring of neural circuits. In *D. melanogaster*, *Dm*Dscam plays a role not only in neuronal self-recognition but also in pathogen recognition. In *A. gambiae*, *Ag*Dscam defends against parasite infections, by binding to pathogens and mediating phagocytosis. This paper highlights the key roles of FREPs and Dscams in the immunity of two major invertebrate groups—mollusks and arthropods—and summarizes the main advancements in current research. These studies not only deepen the understanding of invertebrate immune mechanisms but also lay a solid foundation for future exploration of their potential applications in the biomedical field.

## 1 Introduction

In the biological world, immunological memory refers to the immune system’s capability to retain and remember information about previously encountered pathogens, allowing it to mount a specific response upon subsequent exposure. Immunological memory is thought to be exclusive to jawed vertebrates, achieved through somatic recombination and clonal expansion of lymphocytes (1). Although they lack acquired immunity, invertebrates possess some highly diverse immune molecules that can be used to recognize different antigens, maintain homeostasis, and promote the organism’s adaptability. For example, fibrinogen-related protein (*Bg*FREP) in *Biomphalaria glabrata* (2, 3), Variable chitin-binding proteins (VCBPs) in protochordates (4) and tunicates (5), Down syndrome cell adhesion molecules (Dscam) in arthropods (6–9)—these proteins each have their own corresponding functions, reflecting the unique immune mechanisms that have evolved in invertebrates. In recent years, there has been increasing attention on the highly diverse immune molecules in invertebrates and their roles in immunity as well as other functions.

**Figure 1:**
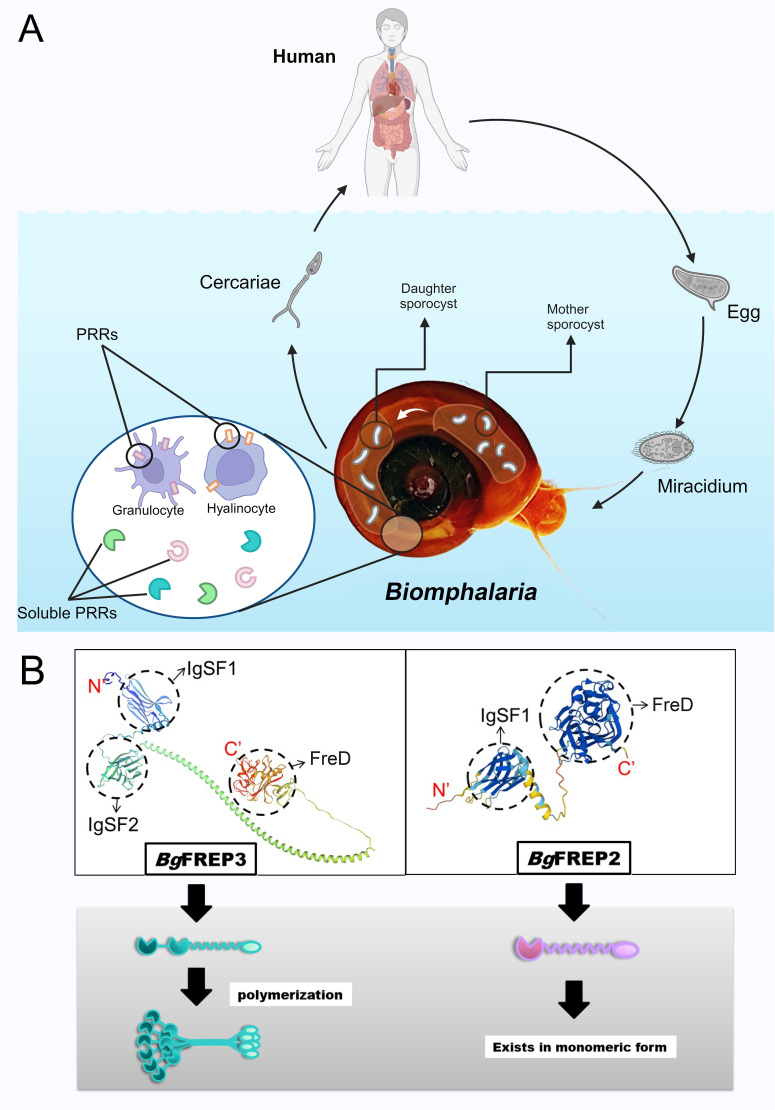
The life cycle of *S. mansoni* and the structural characteristics of the key immune molecules *Bg*FREPs. **(A)** Key stages involved in the life cycle of *S. mansoni* and the immune response of *B*. *glabrata*. Adult schistosomes reproduce within the definitive host (typically a human), and the eggs are excreted through the host’s feces. In a suitable freshwater environment, the eggs hatch into miracidia, which typically invade the snail through the head-foot region. There, they shed their ciliated epidermal plates and develop into mother sporocysts, which then give rise to daughter sporocysts. The daughter sporocysts further replicate, ultimately producing cercariae, which are released from the snail. Once released into the water, the cercariae swim to locate a mammalian host (such as a human). The figure also illustrates the pathogen recognition system of *B*. *glabrata*, which consists of humoral pattern recognition receptors and cellular pattern recognition receptors. **(B)** Structural differences between the *Bg*FREP3 and *Bg*FREP2 proteins. On the left, the 3D modeled molecular structure of *Bg*FREP3 (Uniprot ID: Q8WQX8, URL: Fibrinogen-related protein 3-2 – *B*. *glabrata* (Bloodfluke planorb)UniProtKBUniProt) is shown along with the schematic diagram below (green), which consists of two IgSF domains (IgSF1 and IgSF2) and a FreD domain. These domains are connected by a coiled-coil helical structure, which is hypothesized to facilitate the formation of a multimeric structure. On the right, the 3D modeled molecular structure of *Bg*FREP2 (Uniprot ID: Q95UV9, URL: *Bg*MFREP2 - *Bg*MFREP2 – *B*. *glabrata* (Bloodfluke planorb)UniProtKBUniProt) is shown along with the schematic diagram below (light purple). *Bg*FREP2 has only one IgSF domain, which is connected to the FreD domain by a shorter α-helix structure. According to the literature, it primarily exists in monomeric form. Created with BioRender.com.

Fibrinogen-related proteins, or FREPs, are a family of proteins that contain fibrinogen-related domains (FreD). They are widely distributed across various animal species and exhibit diverse forms and functions. Among them, a unique class of FREPs (*Bg*FREP) with distinct structures exists in the immune system of *B. glabrata* (10). Freshwater snails of the genus *Biomphalaria*, especially *B. glabrata*, play a crucial role as intermediate hosts of *Schistosoma mansoni*, significantly influencing the transmission and geographical distribution of schistosomes (11). *S. mansoni* causes human intestinal schistosomiasis, with symptoms including hepatosplenomegaly, portal hypertension, anemia, and eosinophilia (12). After *S. man*soni miracidium penetrates the intermediate host *B. glabrata*, they transform into mother sporocysts and subsequently develop into daughter sporocysts. This process is a crucial stage in larval infection and the establishment of the parasitic relationship (**Figure 1A**). During this period, they need to successfully defend against or evade the snail’s immune system (12). *B. glabrata* possesses a pathogen recognition system, composed of humoral and cellular receptors, specifically designed to recognize invading schistosomes (13). In humoral immunity, *Bg*FREP acts as a soluble immune factor with lectin properties, classified as a calcium-dependent lectin (10), and plays a key role in the immune response to pathogens. The N-terminus of *Bg*FREP typically contains one or two immunoglobulin superfamily (IgSF) domains (11), while the C-terminus contains a FreD domain. These two regions are connected by an α-helix linker (14, 15), as shown in **Figure 1B**. Molecules with a similar unique structure to *Bg*FREP have also been found in certain other gastropod species (16, 17).

Dscam is widely found across various animal species, particularly in insects, where it was first extensively studied in *Drosophila melanogaster* (*Dm*Dscam) (18). It is a unique neuronal adhesion protein and a member of the immunoglobulin (Ig) superfamily (19, 20). *Ag*Dscam has been identified in *Anopheles gambiae*, where it functions as a hypervariable pattern recognition receptor (PRR) in the immune system (21). Dscam is a complex transmembrane protein with extracellular, transmembrane, and intracellular domains, playing significant roles primarily in nervous system development (20) and the immune system. Dscam has relatively conserved domains in both *D. melanogaster* and vertebrates, including 10 Ig domains, 6 fibronectin type III (FNIII) domains, and a transmembrane domain (19). In *A. gambiae*, each *Ag*Dscam consists of 10 Ig domains, 6 fibronectin repeat domains, and a transmembrane domain (22). The *Dm*Dscam receptor is a typical example of affinity-based binding specificity and is involved in important biological processes (23).

To deepen our understanding of the diversity of FREPs in gastropods and Dscams in insects, and to further comprehend the immune mechanisms in invertebrates, this paper summarizes recent research progress on FREPs and Dscams, particularly on *Bg*FREP in *B. glabrata*, *Dm*Dscam in *D. melanogaster*, and *Ag*Dscam in *A. gambiae*. A comparative analysis of FREPs and Dscams is presented. The article mainly describes the species distribution, molecular structure and diversity, immune-related functions, and other important functions of gastropod FREPs (*Bg*FREP) and insect Dscams (*Dm*Dscam and *Ag*Dscam), along with the latest research developments. Additionally, by comparing FREPs and Dscams—two structurally and evolutionarily distinct immune molecules—this paper highlights their respective roles in invertebrate immunity and their unique mechanisms of pathogen recognition. Such a comparison not only deepens our understanding of innate immune diversity, but also offers new perspectives for biomedical research, particularly in designing novel immune-based therapies and exploring evolutionary principles of immune recognition.

## 2 Fibrinogen-related proteins

### 2.1 FREPs in species

FREP is a broad concept referring to all proteins that contain a FreD domain. In gastropod mollusks, a class of FREPs with unique structural features has been identified in species from certain subgroups of *Heterobranchia* and *Caenogastropoda* (24). These proteins have an IgSF domain at the N-terminus and a FreD domain at the C-terminus, playing an important role in the immune defense mechanisms of these mollusks.

These types of FREPs have been identified in various gastropod mollusks, including *B. glabrata*, *Helisoma trivolvis*, *Lymnaea stagnalis*, *Bulinus truncatus*, *Biomphalaria pfeifferi*, *Helix aspersa*, *Limax flavus*, *Littorina littorea*, and *Aplysia californica* (16). In vertebrates, no FREPs with similar structural features have been found to date. However, proteins containing FreD domains also exist in vertebrates and are involved in immune defense. Examples include those in *Danio rerio* (25), *Oncorhynchus mykiss* (26), *Xenopus laevis* (27), mice (28), chickens (29), and bovines (30).

### 2.2 Exploration of FREPs

Since the 1960s, agglutination molecules have been discovered in the plasma of mollusks (16). In oysters, these lectin-like molecules exhibit opsonization effects. When rabbit red blood cells are incubated in oyster plasma including lectins from *Crassostrea virginica*, the opsonization significantly enhances the phagocytosis of rabbit red blood cells by hemocytes (31). Later, in the 1970s and 1980s, similar lectins were found in the gastropods *B. glabrata* (2) and *Helix pomatia* (32). These lectin-like factors have been shown to participate in non-self-recognition through interaction with carbohydrate targets on the membranes of hemocytes and pathogen-related surfaces in other species (33). In a series of experiments conducted in the mid-1980s, scientists discovered agglutination reactions between snail plasma and schistosome sporocysts. Subsequent studies revealed changes in agglutination factors in snail plasma after exposure to trematode parasites, including upregulation of specific peptides and increased agglutination factor titers (34–38). Scientists isolated and purified agglutination factors from snail plasma and found that these factors are related to schistosome secretion/excretion products (SEP). Further research showed that these agglutination factors are encoded by multiple genes and their expression levels significantly increase after schistosome infection. In 1997, scientists finally discovered and characterized *Bg*FREPs through the analysis of precipitates in snail plasma (10).

As FREP continues to evolve, it reflects the invertebrates’ ability to adapt to environmental pressures. Through gene expansion, domain recombination, and functional diversification, invertebrates can effectively enhance their immune defense capabilities. Research has long shown that after *B. glabrata* is infected with the trematode *Echinostoma paraensei*, its hemolymph contains a lectin composed of a 65 kDa subunit (10). In uninfected snails, three different cDNAs were obtained that were similar to the peptide sequences of the 65 kDa lectin. It was unexpectedly discovered that these encode fibrinogen-related proteins (*Bg*FREP), which also include regions similar to those found in Ig superfamily members (10). This indicates the role of *Bg*FREP in recognizing parasite-derived molecules and provides a paradigm for studying the diversity of non-self-recognition functions in invertebrates (10).

### 2.3 Functional diversity of *Bg*FREPs and their role in immune responses

#### 2.3.1 Variability and polymorphism of *Bg*FREPs

*Bg*FREP plays a vital role in immune recognition and defense during the invasion of trematodes in flatworms, exhibiting high levels of diversity and polymorphism. The diversity and polymorphism of *Bg*FREP are primarily achieved through gene conversion and point mutations, which are essential for covering a wide range of parasite diversity and providing varying levels of infection resistance to snails. This diversity and polymorphism help to understand the evolutionary and adaptive mechanisms between snails and parasites. Recent studies have shown that there are currently 40 known types of the *Bg*FREP family (39), and the mechanisms responsible for generating the diversity of *Bg*FREP at the genomic DNA and mRNA levels have been a focal point of research (3, 11). The *Bg*FREP gene family can serve as a model to study invertebrate immune responses, mechanisms of immune-related gene diversification, and host-parasite interaction molecules (3). Different gene families also exhibit selective immune responses to various pathogens (40). These gene families diversify through alternative splicing, exon loss, and random somatic mutations (41).

Many members of *B. glabrata*, such as *Bg*FREP2, *Bg*FREP3, *Bg*FREP4, and *Bg*FREP12, exhibit somatic diversity (40). Selective splicing is recognized as the most likely mechanism for generating FREP diversity. A large body of transcriptomic research supports this view, revealing different transcript variants of the same *Bg*FREP in mollusks (42). Experiments have shown that transcripts from 20–40 hemocyte pools of *B. glabrata* include different *Bg*FREP sequence modifications (43), confirming that the hemocyte pool is updated during mollusk development and the lineages of *Bg*FREP expressed in these cells also change (40). Pathogen stimulation may enhance the hematopoietic function of *B. glabrata*, thereby promoting the mutation process and increasing *Bg*FREP diversity (44).

Future studies could explore how specific *Bg*FREP variants influence immune response and infection resistance. Investigating how pathogen exposure and environmental factors regulate *Bg*FREP diversity may further illuminate the adaptive mechanisms involved in host-parasite interactions.

#### 2.3.2 Pathogen recognition role

The IgSF domain at the N-terminus and the FreD at the C-terminus of *Bg*FREPs are both considered to be involved in immune recognition (14). Some *Bg*FREPs can form multimers (**Figure 1B**) (14, 45), which affects their biological functions during immune challenges. For example, non-denaturing PAGE analysis shows that *Bg*FREPs in the 65–70 kDa range can form covalent hexamers, with these hexamers observed as complexes of approximately 400 kDa on SDS-PAGE gels (46). These 400 kDa complexes can further associate non-covalently to form complexes observed at around 1600 kDa (46). However, the specific *Bg*FREPs that compose each complex are largely unclear, and research into this facet of *Bg*FREP biology has shown that not every *Bg*FREP can polymerize (16). This capacity for polymerization may depend on their domain architecture, disulfide bond-forming regions, or external stimuli such as pathogen exposure, although the underlying molecular mechanisms remain to be clarified.

The *S. mansoni* polymorphic mucins (*Sm*PoMucs) act as pathogen-associated molecular patterns (PAMPs) to initiate immune recognition and are targets for certain *Bg*FREPs (13). After infection with *S. mansoni*, snails produce large amounts of *Bg*FREPs with lectin-like characteristics. These *Bg*FREPs use their upstream IgSF domains and C-terminal FreD to participate in the response against Schistosoma parasite parasites (47). A study showed that *Bg*FREP2 expression significantly increases in resistant snails, while no such increase was observed in susceptible M-line snails (48). This indicates that two different *Bg*FREP defensive responses may occur in resistant and susceptible snails, leading to different outcomes for the parasites (11).

Despite the immune recognition functions of *Bg*FREPs, certain pathogens have evolve mechanisms to evade these responses. Genomic analyses suggest that parasites may avoid recognition by rapidly evolving their surface proteins (49). Moreover, pathogens can secrete substantial quantities of excretory-secretory products (ESPs), including enzymes and polymorphic mucins, which may further aid in escaping host immune detection (50). Elucidating these evasion strategies will not only deepen our understanding of immune evolution in invertebrates but also inform the development of novel approaches to control infections in susceptible host species.

#### 2.3.3 Synergy

**Figure 2:**
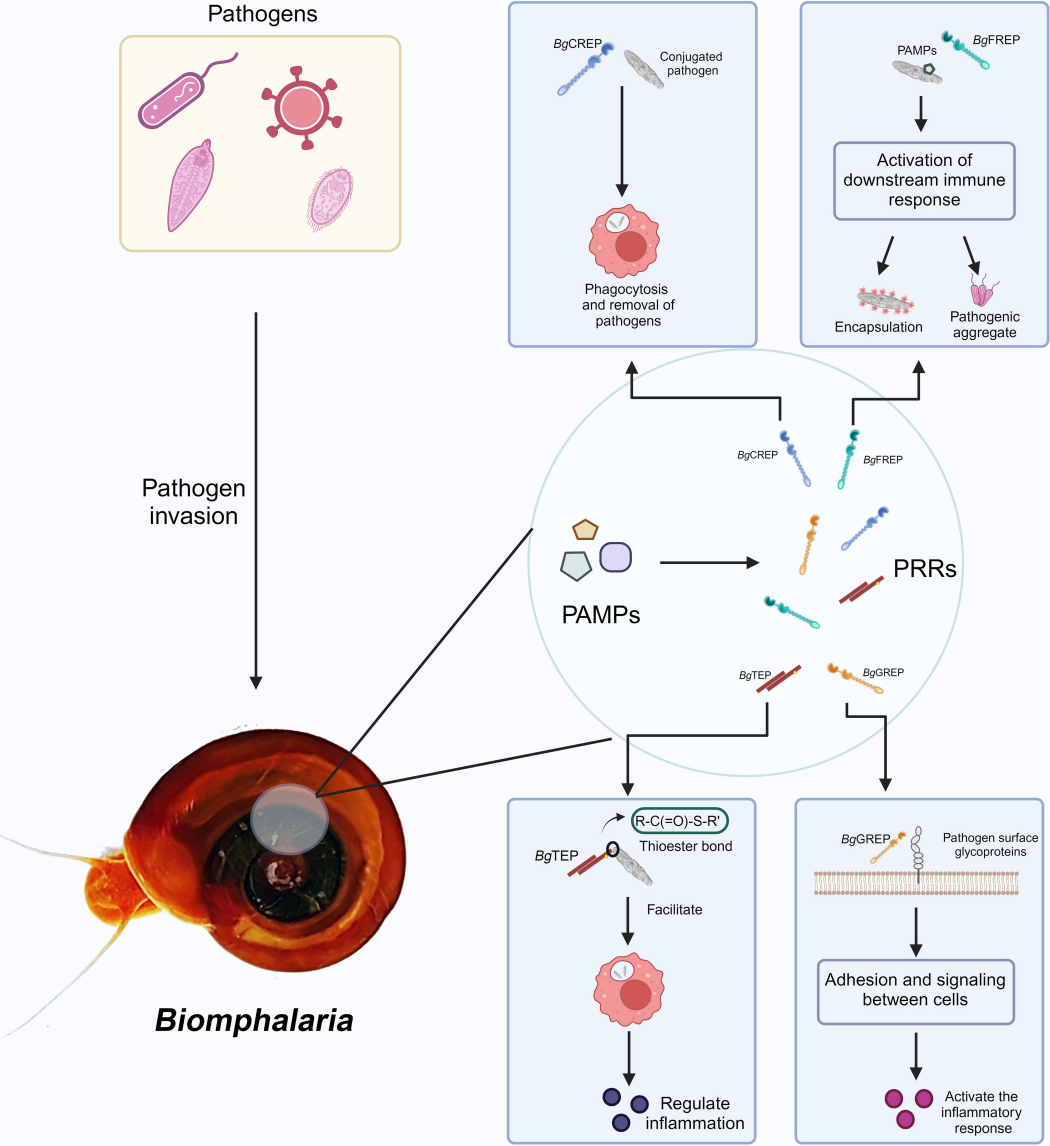
VIgL Family and TEP Family involved in immune recognition and response mechanisms in *B*. *glabrata.* After pathogen invasion into the *B*. *glabrata*, PAMPs are recognized by the snail’s PRRs. The figure shows several key PRRs and their functions: *Bg*CREP binds to pathogens, promoting phagocytosis and aiding in pathogen clearance (54); *Bg*FREP recognizes PAMPs and activates downstream immune responses, including encapsulation and formation of pathogenic aggregates; *Bg*TEP regulates the inflammatory response by promoting the binding of molecules with thioester bonds to targets; *Bg*GREP is involved in cell adhesion and signal transduction, activating the inflammatory response. Created with BioRender.com.

The plasma of *B. glabrata* contains a variety of soluble immune recognition proteins, including numerous PRRs with high polymorphism and diversity. Prominent among these are *Bg*FREPs (49), galectin-related proteins (*Bg*GREPs), C-type lectin-related proteins (*Bg*CREPs) (47), and thioester-containing proteins (*Bg*TEPs) (13, 51, 52). The plasma also contains other types of lectins, endotoxin-binding proteins, and antimicrobial/permeability-increasing proteins, which are capable of recognizing and binding to PAMPs (53) (**Figure 2**). *Bg*FREP, *Bg*GREP, and *Bg*CREPs belong to the variable immunoglobulin and lectin domain (VIgL) family. This family consists of glycoproteins containing IgSF and lectin domains (2, 47). FREPs, CREPs, and GREPs share strong similarity at the amino acid levels, suggesting that these sequences may derive from a shared ancestral gene and/or that these molecules may engage in similar or related biological pathways. The VIgL family is the most significant group of immune lectins targeting schistosome larvae in the plasma (53).

*Bg*TEPs are a key component of the immune system (55), and are secretory glycoproteins distinguished by the presence of a thioester domain featuring a unique intra-chain β-cysteinyl-γ-glutamyl thioester bond (56). TEPs are divided into three main families: the invertebrate-specific TEP family, the complement C3-related family, and the α-2-macroglobulin (A2M) family (57). In freshwater snails, *Bg*TEP is found within a precisely characterized immune complex that includes FREP and *Sm*PoMuc. *Bg*TEP shares several similarities with other invertebrate TEPs, including a domain specialized for A2M receptor binding and a typical thioester motif. Additionally, these TEPs are processed prior to binding to the mucin of schistosome larvae (51). Studies have shown that *Bg*TEP is specifically exhibited in the immune-specialized hemocytes of invertebrates and is released into the hemolymph. *Bg*TEP can bind to the surfaces of different microbes and parasites in either its full-length or processed form. During schistosome infection, immune localization studies have revealed that *Bg*TEP is expressed by only one hemocyte subtype (embryonic cells) (55). This hemocyte subtype is located in the cell protective capsule enveloping the parasite, indicating that it may play an important role in encapsulating and clearing the parasite (55). Whether the specific expression of *Bg*TEP and the splice diversity of *Bg*FREP are involved in immune memory remains unclear, but these mechanisms may contribute to more efficient responses upon repeated pathogen exposure.

*Bg*FREP collaborates with other immune proteins, such as *Bg*TEPs and Biomphalysin, to enhance the immune response. For example, *Bg*FREP2 and *Bg*TEP1 can bind to *S. mansoni* sporocysts, forming immune complexes that increase the production of reactive oxygen species (ROS) by hemocytes, thereby effectively combating the infection. In *B. glabrata* resistant to *Schistosoma* infection, *Bg*TEP1 interacts with *Bg*FREP2 to mediate the recognition of *Sm*PoMucs and enhance resistance to *S. mansoni* infection (13, 14, 55). Immune complexes composed of *Bg*FREP2, *Bg*FREP3, and *Bg*TEP1 can bind to *Sm*PoMucs and promote ROS production by *B. glabrata* hemocytes (53, 58).

#### 2.3.4 *Bg*FREP and immune memory

*Bg*FREP plays a crucial role in the similar immune memory of *B. glabrata* by recognizing and binding to PAMPs, thus stimulating immune responses. The immune function of *Bg*FREP is demonstrated through its differential expression during infection and specific interactions with *Sm*PoMucs (47). Experiments have shown that after an initial infection, *B. glabrata*’s immune system shifts from cellular immune responses, such as phagocytosis and encapsulation, to humoral immune responses, including the secretion of antimicrobial peptides and other immune molecules. After initial infection, *B. glabrata* shows complete protection against subsequent infections with homologous pathogens, which lasts throughout the snail’s entire lifespan (59). Additionally, research has found that this innate immune memory is genotype-dependent, meaning that the protective effect decreases as the neutral genetic distance between the initial and subsequent pathogen infections increases (60). After the first exposure to a pathogen, the polymorphism and expression patterns of *Bg*FREPs can be adjusted and optimized to enhance the response to specific pathogens. Studies have shown that FREPs not only recognize and bind to pathogens during the initial infection but also provide a faster and more effective response during reinfection, including the recognition and binding of pathogens and the activation of immune cells (47).

Future studies could explore whether specific polymorphisms in the *Bg*FREP gene contribute to the differential strength of immune memory responses and whether the expression patterns of *Bg*FREP evolve to optimize pathogen recognition over time. Furthermore, investigating how the immune memory of *B. glabrata* can be modulated by environmental factors or prior exposure to different pathogen strains may provide deeper insights into the mechanisms that govern immune memory in invertebrates. Research into these areas could also reveal potential targets for enhancing immune responses in *B. glabrata* to improve disease resistance.

## 3 Down syndrome cell adhesion molecule in arthropods

### 3.1 Dscams in species

Dscams in Invertebrates are highly diverse cell adhesion molecules that play crucial roles in neural development and immune function. Dscams have been discovered in arthropods (such as *D. melanogaster* (61), honey bees (62), *A. mosquitoes* (63), *Marsupenaeus japonicus* (64), *Scylla paramamosain* (65), *Penaeus monodon (* 66)), annelids (*Hirudo medicinalis* (67)), mollusks (*B. glabrata* (58), *Crassostrea virginica* (68)), as well as *Caenorhabditis elegans* (69), sponges (70). Dscam has also been discovered in vertebrates, including amphibians such as *Xenopus laevis* (71), birds (72), fishes (*Danio rerio* (73)), mammals (humans (74), mice (75)) as well as *Canis lupus familiaris* (69).

### 3.2 Exploration of Dscam

The precursor of Dscam proteins in invertebrates may have existed before the evolution of Bilateria and diploblastic organisms, as seen in cnidarians like *Nemastostella vectensis* and sponges like *Amphimedon queenslandica* (76). Although these organisms lack the typical Dscam structural organization, some of their genes contain highly conserved Ig-like domains with amino acid sequences similar to those of Dscam (76). *N. vectensis* and human DSCAM share similar signaling pathways (7), suggesting that some features of Dscam in complex groups like vertebrates may have evolved early in metazoan evolution. Phylogenetic reconstruction of Dscam molecules across major animal groups indicates that Dscam was initially identified for its role in the nervous system, with its immune properties evolving later (7, 77).

As the Dscam gene evolved across various animal species, it demonstrated the ability to generate diverse isoforms through gene recombination and alternative splicing mechanisms. This diversity plays a crucial role in neural development, immune function, and environmental adaptability. In arthropods, the Dscam gene can generate a variety of different protein isoforms through alternative splicing (77). These isoforms exhibit specificity for various pathogens, enhancing phagocytosis and operating in a manner akin to antibodies (78). Additionally, the Dscam gene regulates connections and recognition between neurons, ensuring the proper formation of neural networks, a mechanism observed in many arthropods (79). In arthropods, unlike in mammals, the Dscam gene exhibits a high level of complexity, suggesting differences in the mechanisms of Dscam function across organisms. While the diversity of the Dscam gene is a unique feature of arthropods, genetic studies on vertebrate DSCAM genes have revealed that the functions of certain molecules in the neural connectivity process are conserved (18).

### 3.3 Diversity and functions of *Ag*Dscam and *Dm*Dscam

#### 3.3.1 The diversity of Dscam

**Figure 3:**
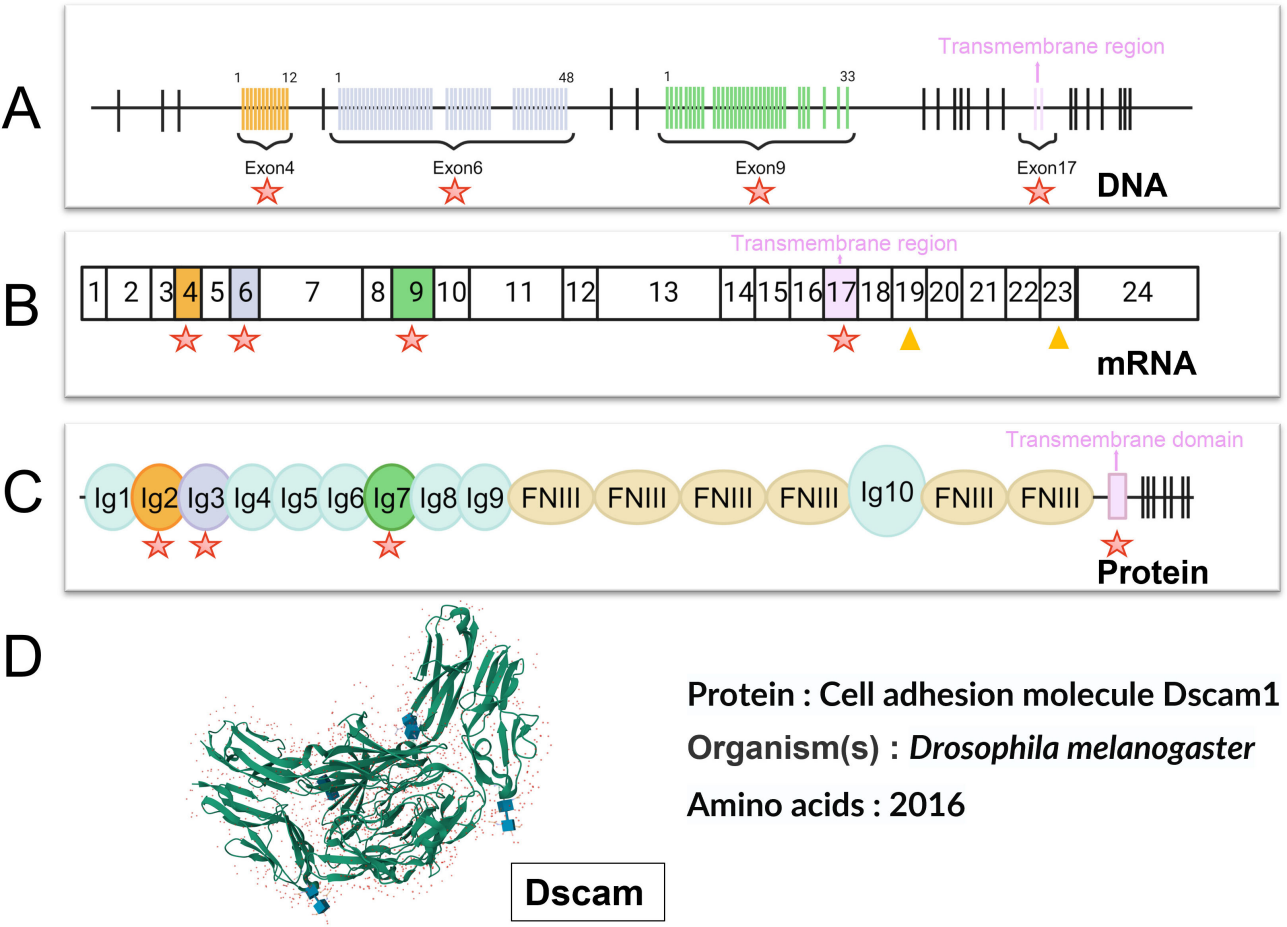
Variable splicing and protein structural diversity of the *Dm*Dscam1 gene. **(A)** *Dm*Dscam1 Gene intro/exon Structure: The *Dm*Dscam1 gene comprises multiple exons, with 20 fixed exons indicated by black vertical lines. Specific exons 4, 6, 9, and 17 (marked with orange, light purple, light green, and pink vertical lines, respectively) are involved in encoding through mutually exclusive splicing. Each of these exons can produce multiple isoforms: exon 4 has 12 variants, exon 6 has 48 variants, exon 9 has 33 variants, and exon 17 has 2 options, resulting in a total of 38,016 (12×48×33×2) potential protein isoforms. Red stars in the diagram highlight the regions of these diverse splicing exons. The pink vertical line represents the transmembrane region. **(B)** *Dm*Dscam1 mRNA organization: All fixed exons are represented by white rectangles. Exons with mutually exclusive splicing capabilities are color-coded to match their DNA counterparts, illustrating their diverse selection in the mRNA. Additionally, internal domain exons 19 and 23 (highlighted by orange triangles in the diagram) can be selectively included or excluded, further increasing the diversity of mRNA isoforms. Considering the four possible combinations of internal domain exons (including exon 19, including exon 23, including both, or including neither) along with the 38,016 mutually exclusive splicing combinations, a total of 152,064 (38,016×4) distinct mRNA isoforms can be generated. Red stars in the diagram indicate the regions of exons with selective diversity. The pink area also represents the transmembrane region. **(C)** *Dm*Dscam1 Protein Structure: The described diversity is reflected in the protein structure, particularly evident in the second (orange), third (light purple), and seventh (light green) immunoglobulin-like domains (depicted as small circles). Detailed Ig protein information can be found on the InterPro website, with domains labeled as Ig1, Ig2, Ig3, Ig4, Ig5, Ig6, and Ig7 (with corresponding numbers: cd20955, cd20953, cd20957, cd20956, cd20958, cd20959, cd20954). Six longer ovals represent the relatively conserved fibronectin type III domains, with detailed FNIII protein information available on the InterPro website (ID: PF18447). The pink vertical bar indicates the transmembrane domain and the black vertical line to its right points to the intracellular region. Red stars in the diagram mark the regions of the amino acid sequences translated from selectively diversified exons. **(D)** *Dm*Dscam1 Protein structure: The figure shows the crystal structure of *Dm*Dscam1 (80). (RCSB PDB ID: 2V5M), more detailed information can be found on the RCSB PDB website (https://www.rcsb.org/structure/2v5m). Created with BioRender.com.

*Dm*Dscam has a notable feature: it generates highly diverse protein isoforms through alternative splicing (**Figure 3**). *D. melanogaster* possesses four genes encoding *Dm*Dscam: *Dm*Dscam1, *Dm*Dscam2, *Dm*Dscam3, and *Dm*Dscam4. Among these, *Dm*Dscam2 and *Dm*Dscam3 undergo a relatively modest scale of alternative splicing, while *Dm*Dscam1 can produce over 38,000 different isoforms through extensive alternative splicing (**Figure 3A**); When considering the independent alternative splicing of internal domain exons, this number increases to 152,064 (**Figure 3C**). Each of these isoforms has a unique extracellular domain (19), formed by distinct combinations of three variable immunoglobulin-like domains, which are linked to one of two alternative transmembrane domains. The variable exon segments are encoded by 12, 48, and 33 variants of exons 4, 6, and 9, respectively, while the transmembrane domain is encoded by two variant exons 17 (81) (**Figures 3A, B**). *Dm*Dscam gene endows each neuron with a unique identity, allowing it to distinguish its own projections from those of other neurons. This self-recognition mechanism is crucial for the proper formation of neural connections in the *D. melanogaster* brain (81).

Similar to *Dm*Dscam, the genomic organization of *Ag*Dscam is also highly complex. It contains 101 exons, including 16 constant exons present in all splice forms and three exon cassettes encoding Ig domains (exon cassettes 4, 6, and 10), which are composed of 14, 30, and 38 alternatively spliced Ig exons, respectively (21). Through alternative splicing, each exon cassette can contribute one Ig exon to a single mature messenger RNA (mRNA), thereby generating potential splice forms with distinct adhesive domains and interaction specificities (21).

Although the structural diversity of Dscam isoforms is well characterized, their role in immune specificity remains unclear. Future research could explore whether specific splice variants are activated by pathogen-specific signals and contribute to immune recognition. This line of investigation could further enhance our understanding of the broader biological roles of Dscam, especially in neurodevelopment and immune function.

#### 3.3.2 Neurodevelopment

**Figure 4:**
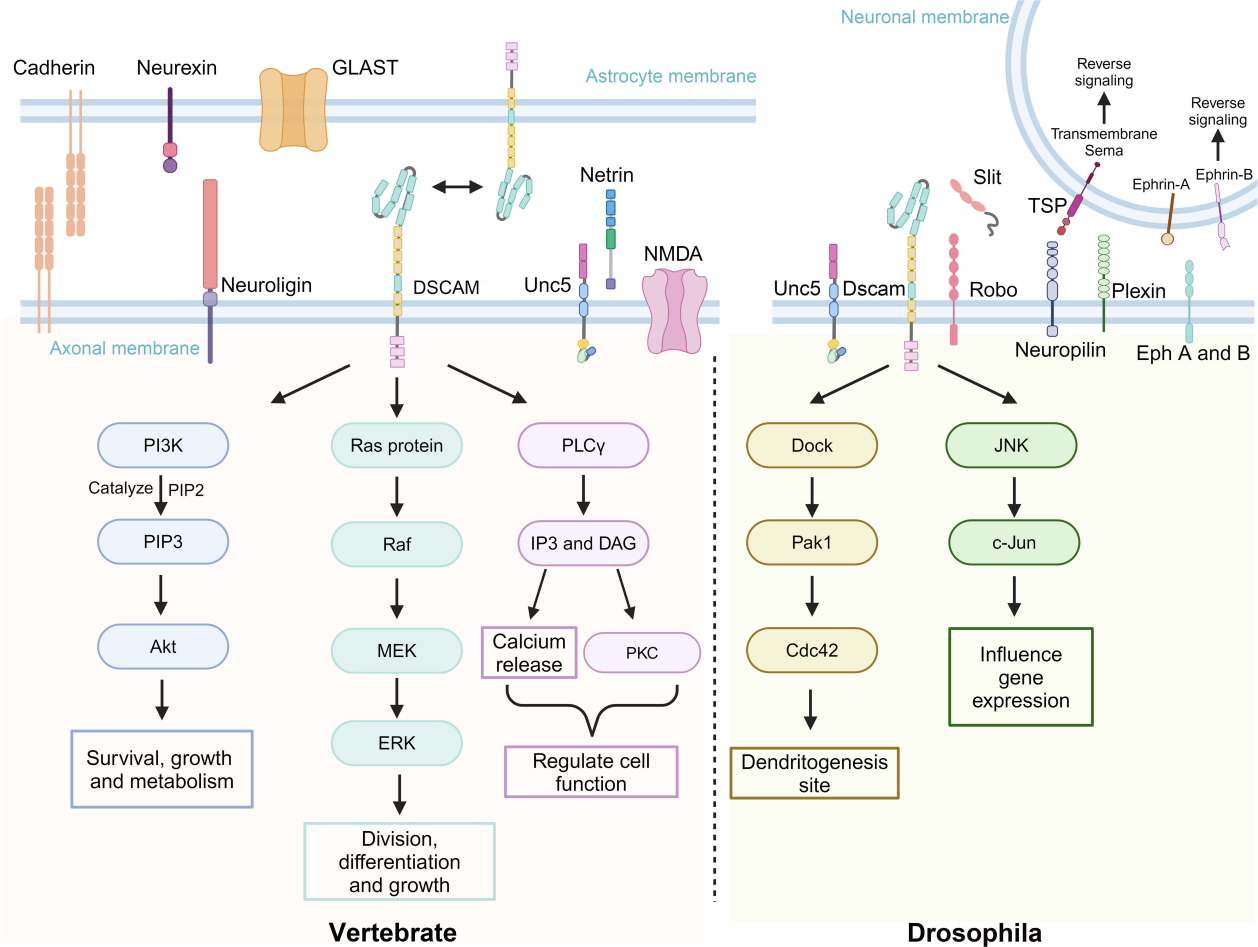
Dscam and its associated transmembrane molecules in signaling pathways of vertebrates and *D. melanogaster.* Dscam exhibits functional conservation and biological diversity across different species, playing a key role, particularly in neural development. The upper part of the image depicts a group of transmembrane molecules associated with Dscam function, whether they directly interact or not. The left side of the lower part illustrates how DSCAM and its related molecules in vertebrates regulate cell growth, differentiation, survival, and function through signaling pathways such as Phosphoinositide 3-kinase - Protein kinase B (PI3K-Akt), Ras - Rapidly accelerated fibrosarcoma - Mitogen-activated protein kinase - Extracellular signal-regulated kinase (Ras-Raf-MEK-ERK), Phospholipase C gamma (PLCγ), and Protein kinase C (PKC). The right side of the lower part shows how *Dm*Dscam in *D. melanogaster* primarily affects gene expression and dendritic development through the Dedicator of cytokinesis - P21-activated kinase 1 - Cell division control protein 42 (Dock-Pak1-Cdc42) and c-Jun N-terminal kinase (JNK) signaling pathways. Created with BioRender.com.

Dscam plays a crucial role in the development of the nervous systems of both *D. melanogaster* and vertebrates, particularly through various signaling pathways (**Figure 4**). The alternative splicing of Dscam not only generates diverse protein isoforms but also stands out due to the complexity of its splicing mechanisms (18). The molecular diversity resulting from this splicing may contribute to the specificity of neuronal connections (20). Additionally, Dock is an adaptor protein containing three Src homology 3 (SH3) domains and one Src homology 2 (SH2) domain and is closely related to the non-catalytic tyrosine-phosphorylated protein (Nck) in mammals (82–84). It plays a crucial role in the guidance and targeting of R-cell axons in *D. melanogaster* (19). *Dm*Dscam interacts with Dock by directly binding to its SH2 and SH3 domains, exhibiting an affinity similar to that of human DSCAM with Dock (20). Studies have also shown that P21-activated kinase (Pak), a p21-activated serine/threonine kinase, acts on Dock’s downstream signaling pathways in adult photoreceptor neurons (85). Genetic research further indicates that *Dm*Dscam, Dock, and Pak work together in the guidance of Bolwig’s nerve, with *Dm*Dscam playing a crucial role in the formation of axonal pathways in the embryonic central nervous system (20).

In *D. melanogaster*, the homologous interactions of the *Dm*Dscam gene require that the interacting isoforms be identical to each other. As previously mentioned, the *Dm*Dscam gene is subjected to extensive alternative splicing, showcasing its diversity (20, 86, 87). This diversity is primarily reflected in the variable regions of the Ig2, Ig3, Ig7 domains and transmembrane domains. Within these regions, homologous interactions are formed through the matching of Ig2, Ig3, and Ig7 domains of identical isoforms (23, 87). Studies have shown that *Dm*Dscam interactions between identical isoforms largely depend on the consistency of the Ig2, Ig3, and Ig7 domains. If there is any variation in these three domains, the interaction cannot occur (88).

*Dm*Dscam plays a unique role in *D. melanogaster* by participating in the mechanisms of “tiling” and “self-avoidance.” The self-avoidance mechanism ensures that the dendrites of a neuron do not intersect with those of other neurons, allowing for precise processing of sensory or synaptic inputs. On the other hand, tiling refers to the phenomenon where certain neurons avoid overlapping with other similar neurons, thereby respecting each other’s territory (89). *Dm*Dscam proteins mediate this tiling process through homologous interactions, allowing these proteins to recognize and prevent adhesion between neurons of the same type or function. This mechanism ensures that dendrites from neurons of the same type can cover sensory and postsynaptic regions with minimal overlap. Additionally, homologous interactions also mediate self-avoidance, where sister neurites from the same neuron repel each other, ensuring uniform coverage of the receptive field and effective processing of sensory information (19). *Dm*Dscam 1 plays a crucial role in the self-avoidance mechanism (90). During development, the axons of mushroom body (MB) neurons bifurcate to form two sister branches, known as the medial and dorsal lobes. Although MB neurons lacking *Dm*Dscam 1 can still bifurcate normally, the process of separating the sister branches is disrupted (19). *Dm*Dscam 2, on the other hand, is involved in the visual system of *D. melanogaster* (91). In this system, neurons include a pair of invariant intermediate neurons, L1/L2, and two pairs of selective neurons, L3. L1 and L2, along with the terminal ends of R cells (R1-R6), form postsynaptic tetrad synapses surrounded by glial cells. This system ensures that axon terminals of the same type of photoreceptor cells remain separated across different ommatidia and do not overlap or make excessive contact within the target area (19).

#### 3.3.3 Immune function

The *A. gambiae* has a complex life cycle, diverse breeding environments, and blood-feeding habits, which expose it to a variety of pathogens, including bacteria, fungi, and viruses (92). *Ag*Dscam can respond to infections by generating splice variant combinations specific to the pathogen challenge (21). Evidence suggests that the specificity can occur at the level of species or even broader pathogen classes, such as bacteria, fungi, and parasites, though strain-level discrimination has not yet been clearly demonstrated (21). The expression levels of these splice variants containing Ig domains (either overexpressed or underexpressed) depend on the type of pathogen encountered by the host cells. This specificity is demonstrated by the observation that two species of Plasmodium, which cause malaria, as well as different bacterial pathogens such as *Escherichia coli* and *Staphylococcus aureus* (21), induce significantly different splice variant combinations in adult *A. gambiae* (93). When *Ag*Dscam is transiently silenced, the *A. gambiae*’s ability to resist bacterial and malaria parasite infections is significantly impaired. *Ag*Dscam can bind to bacteria through specific splice variants and mediate phagocytosis, thereby contributing to pathogen defense (21). Previous studies have shown that the immune response in mosquitoes is significantly regulated by the Immune deficiency (IMD)and Toll-like receptor (Toll) pathways. Activation of the immune response mediated by the IMD pathway is more effective in inhibiting *Plasmodium falciparum* infection, while activation of the Toll pathway exhibits a more specific defense against *Plasmodium berghei* (92–94). Additionally, the IMD and Toll pathways also regulate the selective splicing of *Ag*Dscam. To confirm this, a study analyzed the transcriptional changes of *Ag*Dscam under basal conditions or upon immune activation by lipopolysaccharide (LPS) and peptidoglycan (PGN). The results showed that activation of either the IMD or Toll pathway significantly altered the mRNA levels of *Ag*Dscam’s variable Ig domains, with significant differences compared to the control group (22). *Ag*Dscam can generate different splice variant pools in response to at least eight different immune elicitors, thereby enabling pathogen-specific defense (21, 22).

**Figure 5:**
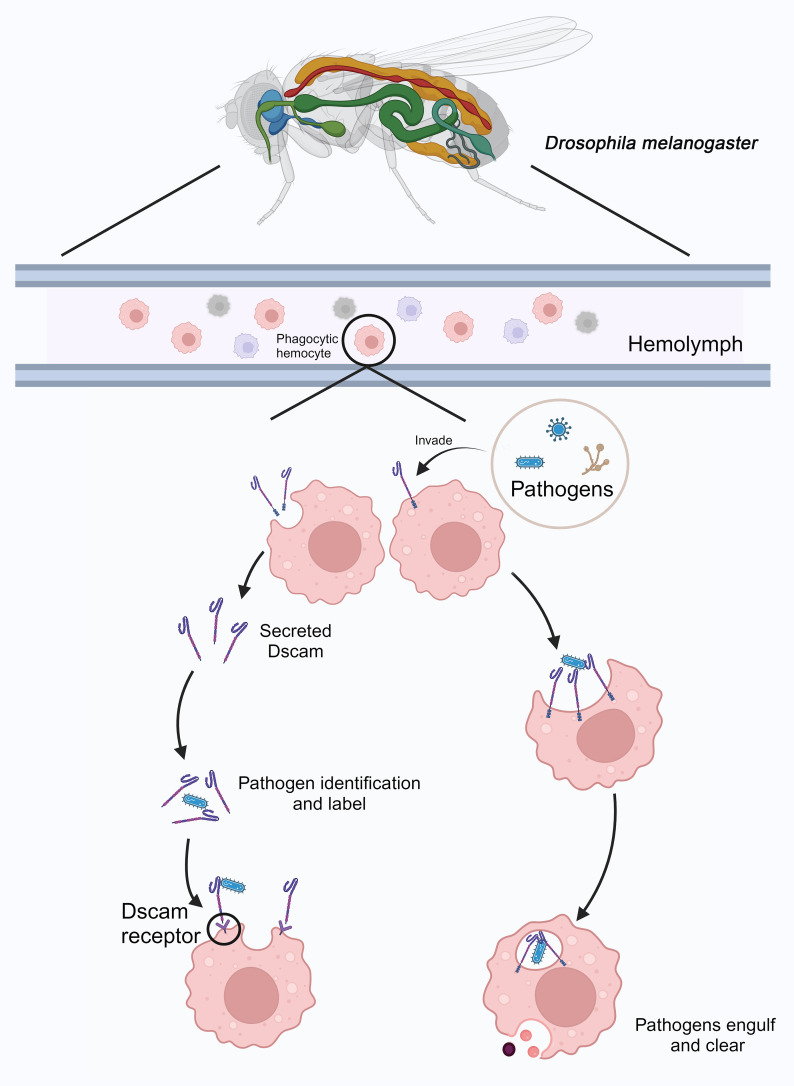
Immune response mediated by *Dm*Dscam in *D. melanogaster.* In *D. melanogaster*, phagocytic hemocytes patrol the hemolymph. When pathogens invade, some of these phagocytic cells secrete *Dm*Dscam molecules to recognize and tag the pathogens. Pathogens labeled with *Dm*Dscam are then identified, bound, and cleared by other phagocytic cells through the *Dm*Dscam receptors. Additionally, *Dm*Dscam on the surface of certain phagocytic hemocytes can directly recognize and bind to pathogens, leading to their phagocytosis and elimination. Created with BioRender.com.

*Dm*Dscam is involved in pathogen recognition and is associated with the phagocytosis and clearance functions of immune cells in *D. melanogaster*. A potential model for pathogen immune responses is illustrated in **Figure 5**. *Dm*Dscam is a single-pass transmembrane receptor that not only functions in axonal pathways but is also expressed in phagocytic hemocytes (95). The *Dm*Dscam gene encodes proteins containing immunoglobulin (Ig) domains through alternative splicing, potentially generating thousands of protein isoforms (Dscam Hypervariable, Dscam-hv). This splicing process involves mutually exclusive selection of exons encoding half of the Ig2 domain, half of the Ig3 domain, and the entire Ig7 domain (96).

Recombinant *Dm*Dscam proteins can bind to *E. coli*, and hemocytes extracted from *Dm*Dscam gene-knockdown *D. melanogaster* larvae or S2 cells (a *D. melanogaster* cell line) pre-incubated with *Dm*Dscam antibodies show reduced bacterial phagocytic activity, indicating that *Dm*Dscam may function as a receptor or regulatory factor in phagocytes (96). Although increasing evidence suggests that *Dm*Dscam-hv might act as a pathogen-specific recognition molecule, with different *Dm*Dscam-hv variants recognizing different pathogens and participating in immune memory similar to adaptive immunity in vertebrates, its role in invertebrate immunity remains controversial (96). For example, some studies have shown that short-term expression of *Dm*Dscam genes in *D. melanogaster* larvae hemocytes and fat bodies does not significantly change after exposure to *E. coli*, *Pseudomonas fluorescens*, and *Bacillus thuringiensis* (97). Similarly, other research has found that the overall splice variant diversity of Dscam1 in *D. melanogaster* does not significantly change after early exposure to bacteria such as *E. coli* (98), while the effects of viral or fungal infections on this diversity remain to be fully elucidated. However, in *A. gambiae*, the diversity of Ig2 and Ig3 variants of *Ag*Dscam increases after pathogen infection (98, 99). This dynamic shift in splice variant usage may also reflect an aspect of immune response plasticity (100). Therefore, further research is needed to validate the immune functions of Dscam. Future studies could test the hypothesis that specific Dscam isoforms are selectively induced by distinct pathogens and mediate differential immune responses through regulated alternative splicing.

Despite its role in pathogen recognition and immune defense, some pathogens have evolved strategies to evade Dscam-mediated immune responses. These strategies may include antigenic variation, whereby pathogens alter their surface proteins to avoid detection (21); or suppression of the host’s complement-like immune system through their own surface molecules (101). A deeper understanding of these evasion mechanisms is crucial for elucidating the complexities of host-pathogen interactions and enhancing immune defense strategies in invertebrates.

### 3.4 Dscam and disease

In *D. melanogaster*, the absence of *Dm*Dscam leads to abnormal neural development (20), increased susceptibility to infections, and reduced phagocytic activity (9). Conversely, overexpression of *Dm*Dscam can affect the intestinal nervous system of *D. melanogaster*. Studies have found that overexpression of *Dm*Dscam1 in the intestinal nervous system of *D. melanogaster* leads to excessive growth of the foregut and hindgut neurons, reducing the efficiency with which the larvae clear food from the gut (102). Additionally, a systematic study has tested the effects of *Dm*Dscam1 on dendrite growth and spacing in eight different types of central nervous system neurons in *D. melanogaster*. It was observed that knockdown of *Dm*Dscam1 resulted in severe dendritic clumping and reduced length in output glutamatergic and aminergic neurons (103).

Abnormal expression of DSCAM is associated with human diseases such as Down syndrome (DS), autism, and certain immune disorders. Research based on the location of DSCAM on chromosome 21, its specific expression in the central nervous system and neural crest, and its involvement in neural migration, differentiation, and synaptic function suggests that DSCAM is involved in neural differentiation and is related to defects in the central and peripheral nervous systems observed in DS (104). Additionally, some studies have concluded that DSCAM may act as an inhibitory factor that prevents premature maturation of the spinal cord and excessive glutamatergic transmission, with its deficiency potentially leading to autism-like behaviors (105).

## 4 Summary and outlook

In invertebrates, FREPs and Dscams are two important immune molecules. Both contain immunoglobulin domains and are closely related to development and the innate immune response. They each exhibit unique immune functions and mechanisms. *Bg*FREP is a soluble pattern recognition receptor in *B. glabrata*, which enhances the immune response to parasites (such as schistosomes) by binding PAMPs and forming multimers. Its diversity primarily arises from gene conversion and point mutations. In contrast, Dscam has been mainly studied in *D. melanogaster* and *A. gambiae*. It is a transmembrane protein containing immunoglobulin-like domains, which generates specific isoforms to respond to pathogen infections through an extremely complex process of selective splicing. In *D. melanogaster*, *Dm*Dscam is more involved in pathogen recognition and phagocytosis regulation, while in *A. gambiae*, *Ag*Dscam’s function is more focused on binding to pathogens and blocking parasite development through the defense mechanisms of midgut epithelial cells (106). This functional difference reflects the adaptive evolutionary strategies of the two model organisms in responding to different immune challenges.

The comparison between *Bg*FREP and Dscam in terms of amplification and selection reveals the diversity and flexibility of the invertebrate immune system. *Bg*FREP generates a broad antigen recognition capacity through gene recombination and somatic diversification, adapting to the complex and dynamic parasitic environment. In contrast, Dscam in *D. melanogaster* and *A. gambiae* generates tens of thousands of different isoforms through selective splicing, reflecting the high precision of immune function. This mechanistic difference not only highlights the flexibility of the invertebrate immune system but also suggests that PRRs may evolve highly specialized functions in different species. Furthermore, regarding the specificity of PRRs, studies in some invertebrates indicate that these molecules can generate specific receptor repertoires to respond to different pathogens (107). This phenomenon is particularly evident in the Dscam of *D. melanogaster* and *A. gambiae*, further challenging the traditional paradigm that invertebrate immune responses are ‘simple’ and ‘lacking specificity’. These findings suggest that invertebrates may form complex immune memory mechanisms through genetic diversity and selective splicing, offering new directions and insights for research in the field of immunology.

Future research could further explore the regulatory networks and molecular mechanisms of FREP and Dscam in invertebrates. For example, do FREP and Dscam exhibit similar regulatory mechanisms across different species? Do other mollusks also possess polymorphism-generating mechanisms similar to *Bg*FREP? Additionally, through modern molecular biology techniques such as single-cell sequencing and structural biology, it will be possible to gain deeper insights into the distribution and dynamic functions of these molecules at the cellular and tissue levels. At the same time, expanding the study of *Bg*FREP and Dscam to other invertebrates may reveal new types of PRRs or expand existing functional patterns. These studies will lead to new breakthroughs in the research of invertebrate immune mechanisms and contribute to innovations in biomedical and biotechnological fields.
